# Nutlin‐3a as a novel anticancer agent for adrenocortical carcinoma with *CTNNB1* mutation

**DOI:** 10.1002/cam4.1431

**Published:** 2018-03-13

**Authors:** Wen Hui, Shenghua Liu, Jie Zheng, Zujun Fang, Qiang Ding, Chenchen Feng

**Affiliations:** ^1^ Department of Urology Huashan Hospital Shanghai 200040 China; ^2^ Fudan Institute of Urology Shanghai 200040 China

**Keywords:** Adrenocortical carcinoma, *CTNNB1*, MDM2, Nutlin‐3a, *TP53*

## Abstract

Adrenocortical carcinoma (ACC) is a rare malignancy, and *CTNNB1* is frequently mutated in ACC. Our study aims to screen for effective agents with antineoplastic activity against ACC with *CTNNB1* mutation. In‐silico screening of the Genomics of Drug Sensitivity in Cancer (GDSC) database was conducted. Drug sensitivity in cells with *CTNNB1* mutation was analyzed and further in vitro and in vivo studies were performed using the compound. Only one compound, Nutlin‐3a, an MDM2 inhibitor, was significantly sensitive in 18 cancer cells with *CTNNB1* mutation. Further analysis of the 18 cells revealed no significant efficacy between cells with both *CTNNB1 and TP53* mutations indicating concomitant *TP53* mutation did not impact on drug efficacy. We verified that Nutlin‐3a inhibited cellular proliferation in ACC cell line NCI‐H295R which harbored *CTNNB1* mutation but not in SW13 cells which did not. Nutlin‐3a induced cell apoptosis and G1 cell‐cycle arrest in NCI‐H295R cells. Nutlin‐3a also decreased cellular migration and inhibited epithelial‐to‐mesenchymal transition (EMT) process in terms of EMT index. Nutlin‐3a resulted in decreased β‐catenin level independent of p53 level in NCI‐H295R but not SW13 cells. We also evaluated the effect of Nutlin‐3a on hormonal secretion of NCI‐H295R cells and found it resulted in decreased levels of cortisol, androgen, and progesterone. Nutlin‐3a treatment inhibited ACC tumor growth with no observed toxicity in mice in vivo. Our study has revealed that Nutlin‐3a potently inhibits ACC with *CTNNB1* mutation. How p53/MDM2 axis coordinates with Wnt/beta‐Catenin signaling in ACC warrants further study.

## Introduction

Adrenocortical carcinoma (ACC) is a rare endocrine malignancy with an annual incidence ranging from one to two case per million people 1. Clinical stage classification proposed by the European Network for the Study of Adrenal Tumors (ENSAT) is recommended as staging remains the most solid prognostic parameter 2. The ENSAT stage, unlike conventional staging systems that solely encompass tumor extension, includes hormonal workup as a whole.

According to ENSAT guideline, complete surgical resection is the only curative method to treat ACC and management of hormonal excess is majorly dependent on adrenolytic agents. However, most of the cases are locally invasive or have metastasized to distant organs at the time of diagnosis. For advanced ACC, combination of cytotoxic drugs and mitotane was the recommended but the poor therapeutic efficiency and severe side effects limit clinical utility 3. The prognosis of ACC is still poor and the overall 5‐year survival rate is merely 25–50% 4. Thus, developing new agents to improve the therapeutic effects is urgent.

For decades, extensive efforts have been made on the unveiling of the genomic characteristics of ACC 5. *CTNNB1* is frequently mutated in ACC and is identified as one of the driver mutations 6, 7. *CTNNB1* encodes β‐catenin which is a subunit of the cadherin protein complex and acts as an intracellular signal transducer on the activation of Wnt signaling. The activating mutation or overexpression of *CTNNB1* leads to the activation of Wnt/β‐catenin pathway and is associated with tumorigenesis of ACC 8. Contemporary multilevel next‐generation sequencing has not only revealed a series of genetic alterations that consolidate the activated Wnt signaling in ACC, but also strengthened the predominant role of β‐catenin in Wnt activation 9, 10. Therefore, targeting *CTNNB1* could be potential therapeutic strategy for the treatment of ACC.

In our study, we aim to explore the online Genomics of Drug Sensitivity in Cancer (GDSC) database to screen drug sensitivity in cells with *CTNNB1* mutation. Further in vitro and in vivo studies were performed using the compound.

## Materials and Methods

### Data mining and analysis of TCGA database

An in‐silico reproduction using TCGA database was performed in this study as previously reported 10, 11. The TCGA adrenocortical carcinoma (Provisional) database was selected on the cBioPortal online platform 12, 13. Case set within “Tumors with sequencing and CNA data” were selected, in which the tumor and clinical profiles were all complete in all 84 patients, whereas mutation and CNV was complete in 88 patients. Cases with *TP53* and *CTNNB1* alteration, including mutation and CNV, were queried using the OncoPrint function. The relationship between *CTNNB1* mutation or expression and clinical characteristics, especially hormone execration, was analyzed.

### Data mining and analysis of GDSC database

The GDSC database encompassed the sensitivities and genomic profiles of a variety of cancer cell lines to a number of compounds. We started by searching compounds with significant selectivity for *CTNNB1* mutations on the online visual platform (http://www.cancerrxgene.org/). The selected compounds were then tested for TP53 mutations. A search through the pan‐cancer cell line strategy was applied. With the aim to improve cancer treatments by discovering therapeutic biomarkers that can be used to identify patients most likely to respond to anticancer drug, the GDSC database harbors screening >1000 genetically characterized human cancer cell lines with a wide range of anticancer therapeutics. Our search thus included all cancer cell lines available with genetic status of *CTNNB1* and *TP53*. The volcano plots and scatter plots were generated and computed via the GDSC online platform.

### Cell culture

Two ACC cell line H295R and SW13 were purchased from American Type Culture Collection (ATCC). Cells were cultured in RPMI‐1640 medium (Life Technologies) containing 10% FBS, 100 units/mL penicillin, and 0.1 mg/mL streptomycin. Cells were maintained in an atmosphere of 5% CO_2_ at 37°C.

### Lentiviral RNA overexpression

Lentivirus encoding *CTNNB1* shRNA was generated by GeneChem Company (Shanghai, China). Lentivirus expressing scramble shRNA was used as negative control. Cells were plated in twelve‐well plates (1 × 10^5^ cells per well), transduced with 5 MOI lentiviral particles (using 8 *μ*g/mL hexadimethrine bromide [Sigma]), and incubated at 37°C, 5% CO_2_. Overexpression of CTNNB1 in stable transfected cells was confirmed by qPCR.

#### RNA interference

The H295R cell line was subject to *CTNNB1* knockdown (KD) using shRNAs. The ORF sequences for *CTNNB1*‐KD were chosen from TRC (TRCN0000314990 for shRNA#1 and TRCN0000350477 for shRNA#2). Viral transduction was performed according to standard protocol 14. Briefly, viral particles were prepared in 293T cells. Starved cells were transduced with equal titers of enveloped particles in OptiMEM media (Thermo) supplemented with 1 *μ*g/mL polybrene (Sigma, Deisenhofen, Germany) for 24 h. Puromycin was used for selection.

#### Quantitative RT‐PCR

Expression of *CTNNB1* was quantified using Real‐time RT‐PCR, per established protocol 15. Primers for *CTNNB1* were as follows: Forward 5′‐ AAA GCG GCT GTT AGT CAC TGG ‐3′; Reverse 5′‐ CGA GTC ATT GCA TAC TGT CCA T ‐3′. We used *GAPDH* primers for internal reference 15. After conversion of extracted mRNA to cDNA, real‐time PCR procedure with SYBR Green Premix Ex Taq (TaKaRa) in a 20‐*μ*L system was run on ABI 7500n (Applied Biosystems, Forster City, CA). For each sample, the average value of threshold cycle was normalized to *GAPDH* level with the formula, 2^−∆∆Ct^.

### Cell proliferation assay

Cell viability in each group was measured using MTT. The cells were seeded into 96‐well plates at a density of 2 × 10^4^ cells/well, cultured overnight, and treated as mentioned above. Next, 10 *μ*L of 5 mg/mL MTT was added to each well. After 4 h further incubation, the optical density at 490 nm of each well was measured with a microplate reader. Cell proliferation ability was analyzed daily for five consecutive days.

### Cell cycle assay

Cells were plated in 25 cm^2^ flasks and incubated overnight. Then the cells were collected and fixed in precold 70% ethanol for 1.5 h at 4°C. After fixation, the cells were washed in PBS again and centrifuged for 5 min at 1000 rpm. The PBS was discarded, and propidium iodide (PI) was added to a final concentration of 50 *μ*g/mL in dark at 4°C for 30 min. Flow‐cytometric analysis was performed on the FACSCalibur flow cytometer (Becton Dickinson, Franklin Lakes, NJ). The cell cycle was analyzed using Cell Quest software.

### Cell apoptosis assay

Cells were plated in 25 cm^2^ flasks and incubated overnight. Then the cells were collected and adjusted with staining buffer at a density of 10^5^–10^6^ cells/mL. After incubation with 5 *μ*L of Annexin V‐FITC and 10 *μ*L of PI in dark for 15 min, cells were analyzed by flow cytometry (Becton Dickinson). Apoptosis cells were recognized as high Annexin V fluorescence signal with low PI signal. The percentages of apoptotic cells were calculated by data from FACS analysis.

### Migration assay

In vitro migration assays were performed according to standardized protocol. Briefly, cells were cultured in the upper chamber with serum‐free media that were coated with matrigel for migration assays, respectively. Then culture medium was placed in a 24‐well plate filled with medium with 20% FBS. The cells were allowed to migrate through a porous surface to the bottom of the membrane. Then the migrated cells on the bottom of the membrane were fixed and stained with crystal violet. Cells were transferred to a 96‐well plate to measure the absorbance at 570 nm using a microplate reader. All the assays were repeated at least three times.

### Western blotting

Protein levels were quantified by Bradford assay. The protein sample was diluted, heated for denaturation, and then subjected to dodecyl sulfate polyacrylamide gel electrophoresis (SDS‐PAGE) and transferred onto polyvinylidene fluoride membranes (PVDF, Millipore). The membrane was blocked in 0.1% Triton X‐100 and 5% nonfat milk powder in phosphate‐buffered saline for 1 h at 4°C. Primary antibodies against p53 (Abcam) and Mdm2 (Abcam) were then added and membranes were kept incubating at 4°C overnight. Corresponding secondary antibodies were applied followed by electrochemiluminescence (ECL) processing.

### Hormone measurement

Hormones were measured using Multi‐Spot 96 HB 4‐Spot Custom Steroid Hormone Panel (Mesoscale Discovery). The custom cortisol assay added to the available 3‐plex encompassing progesterone and testosterone 16; 50 *μ*L of spent culture medium was run per well in duplicate, and values were calculated based on a standard curve. Values are represented as percent of DMSO control.

### EMT index

Reporter constructs pVFir and pERuc used to quantitate EMT were constructed by fusing the human *VIM* promoter region (nt −1629 to −47 relative to the translation start site) and the human *CDH1* promoter region (nt −1115 to −65) to the coding region of the firefly luciferase and the Renilla luciferase, respectively 17. These chimeric genes were then cloned into the SIV‐based lentivector pSIV‐gaMES4SA. Viral particles pseudotyped with the VSVG envelope were produced as described previously. Forty‐eight hours after infection with pVFir and pERuc, expression of the firefly and Renilla luciferase expression was measured with a microplate luminometer (Luminoskan Ascent, Labsystems) using the Dual‐Glow luciferase assay system (Promega). The EMT index (EMTi) was calculated as the ratio of firefly to Renilla luminescence.

### ACC xenograft models

Twenty‐four male BALB/c nude mice at 6 weeks of age were bred in SPF (special pathogen‐free) grade laboratory. Mice were randomly divided into four groups (H295R and SW13 cells; treatment vs. control). A total of 10^7^ cancer cells resuspended in 100 mL of PBS were injected subcutaneously at the left axilla of each mouse. Tumors became perceptible at approximately 4 mm in diameter on approximately day 7. Thereafter, intraperitoneal injection of Nutlin‐3a at 25 mg/kg/day or control (PBS) at equivalent volume was given. All mice were sacrificed on day 35, and tumors were extracted. Tumor size was calculated with the formula, volume = length × width^2^/2. All experimental protocols were approved by Institutional Review Board of Department of Laboratory Animal Science of Fudan University.

## Results

### Mutant *CTNNB1* is associated with hormone excess

**Figure 1:**
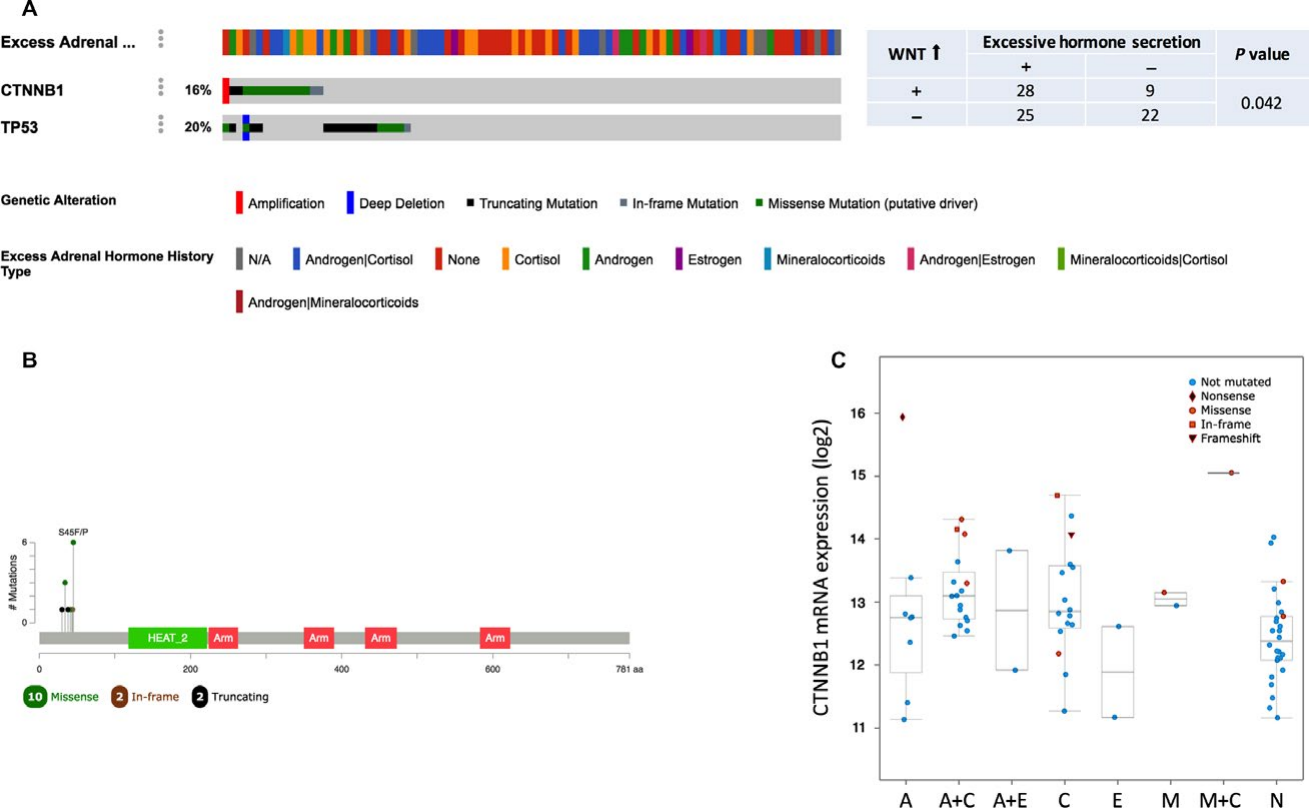
*CTNNB1* was frequently mutated in ACC and was associated with excessive hormone secretion. (A) Reproduction of The Cancer Genome Atlas (TCGA) database showing genetic alteration status of *CTNNB1* and TP53 gene in ACC patients; Changes in *CTNNB1* gene sets were associated with excessive hormone secretion (*P* = 0.042). (B) The mutation locus of site of *CTNNB1* was relatively concentrated. (C) High *CTNNB1* gene expression level was associated with hormone secretion history.

As adrenocortical carcinoma is remarkable for its excessive hormone‐producing ability, we analyzed the characteristics of hormone secretion based on TCGA ACC dataset. Among the 88 cases, almost half of the patients had a history of excessive hormone. There were 39 cases with hormone excess among whom 17 with excessive cortisol, 8 with androgen, 2 with estrogen, and 2 with mineralocorticoids, respectively, while 24 cases had excess of two hormones. *TP53* and *CTNNB1* were altered in 20% and 17% of the cases, respectively (Fig. 1A). As Wnt pathway activation was critical in ACC tumorigenesis, *CTNNB1* overexpression and its association with hormone was analyzed. Wnt activation was found to be significantly associated with hormone excess (*P* = 0.046) (Fig. 1A and C). The mutation sites of *CTNNB1* were clustered on the N‐terminal segment of β‐catenin (Fig. 1B).

### Cancer cells with CTNNB1 mutation are sensitive to Nutlin‐3a

**Figure 2:**
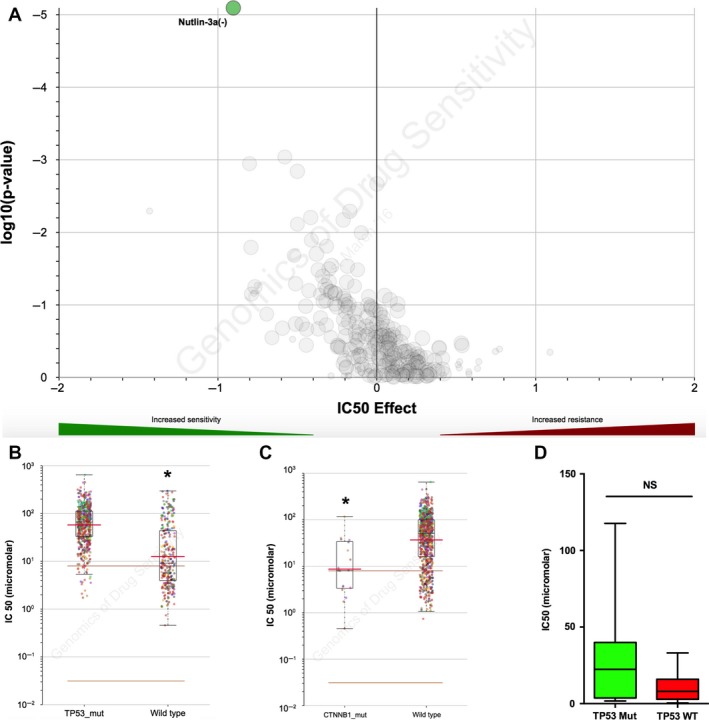
Cancer cell lines with *CTNNB1* mutation were sensitive to Nutlin‐3a, irrespective of TP53 mutation status. (A) Reproduction of the Genomics of Drug Sensitivity in Cancer (GDSC) database. Volcano plotting showing compounds marked in green as sensitive in the setting of pan‐cancer cells with *CTNNB1* mutations. (B) Scattered plotting showing cells with TP53 mutations were resistant to Nutlin‐3a, while (C) cells with *CTNNB1* were sensitive to Nutlin‐3a. (D) TP53 mutation status did not affect selectivity for Nutlin‐3a among cell lines harboring *CTNNB1* mutation. (*P < 0.05).

Current pharmaceutical agent for ACC is lacking. Exploiting potent tumor inhibitor is therefore a must. As *CTNNB1* was the truncal gene in ACC, we explored compounds in GDSC database with significant selectivity to cancer cells harboring *CTNNB1* mutation. Only one compound, Nutlin‐3a, showed significant selectivity for *CTNNB1* mutation (Fig. 2A). Nutlin‐3a inhibits the interaction between MDM2 and p53, restoring the p53‐mediated tumor suppression pathway. Therefore, *TP53*‐mutated cancer cells demonstrated resistance to Nutlin‐3a (Fig. 2B). Interestingly, when those cases were grouped using *CTNNB1* mutation status, Nutlin‐3a still showed significant selectivity for *CTNNB1*‐mutant cases (Fig. 2C). To investigate whether *TP53* mutation could interfere with Nutlin‐3a's selectivity, we further stratified *TP53* mutation among *CTNNB1*‐mutated cell lines and found no statistical power (Fig. 2D). Here we showed that MDM2 inhibitor, Nutlin‐3a, conferred selective inhibition in cancer cells with *CTNNB1* mutations, regardless of *TP53* mutation status.

### ACC cells with *CTNNB1* mutation is sensitive to Nutlin‐3a

**Figure 3:**
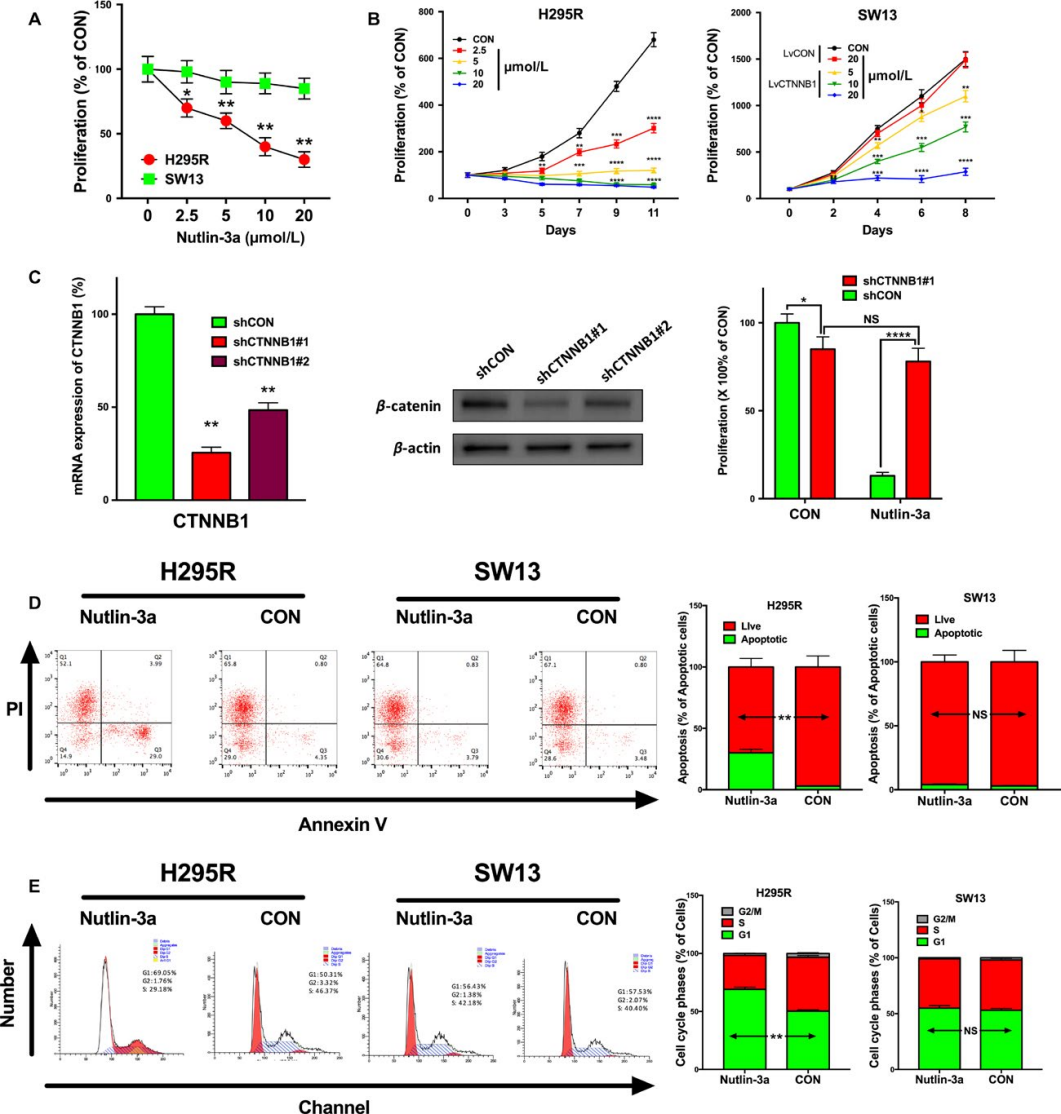
Nutlin‐3a selectively inhibited ACC cell with *CTNNB1* mutation. (A) Nutlin‐3a selectively inhibited H295R which harbor *CTNNB1* mutation instead of SW13 which does not. (B) After *CTNNB1* gene was overexpressed, SW13 could be inhibited by Nutlin‐3a as H259R. (C) Cell apoptosis assay shows Nutlin‐3a significantly induces apoptosis in H259R instead of SW13 (D) Cell cycle assay shows Nutlin‐3a significantly causes G1/S arrest in H259R instead of SW13. (*P < 0.05, **P < 0.01).

We then examined the effects of Nutlin‐3a in vitro. ACC cell line NCI‐H295R and adrenocortical small‐cell carcinoma cell line SW13 were used. The COSMIC database demonstrated that H295R cells harbored activating mutation in *CTNNB1* and wild‐type *TP53* while SW13 cells harbored mutated *TP53* and wild‐type *CTNNB1*. Therefore, we treated H295R and SW13 with Nutlin‐3a at the indicated concentration. Nutlin‐3a inhibited proliferation of H295R in a dose‐dependent and time‐dependent manner. Nevertheless, the inhibition effects of Nutlin‐3a at the same concentration on SW13 were minimal. However, the inhibition was restored in SW13 cells when *CTNNB1* was overexpressed with lentivirus (Fig. 3A–B). Silencing of *CTNNB1* in H295R cells effaced the inhibitory effect of Nutlin‐3a at 10 *μ*mol/L on day 9 (Fig. 3C). Interestingly, we found that CTNNB1‐KD itself also inhibited cell proliferation to a lesser extent (Fig. 3C). We then explored apoptotic effect of Nutlin‐3a using flow cytometry and found an increase in the percentage of apoptotic cells in the Nutlin‐3a‐treated cells only in H259R, but not SW13 cells (Fig. 3D). Similarly, Nutlin‐3a induced G1/S arrest in H259R but not in SW13 cells (Fig. 3E).

### Nutlin‐3a inhibits multiple properties of ACC cells

**Figure 4:**
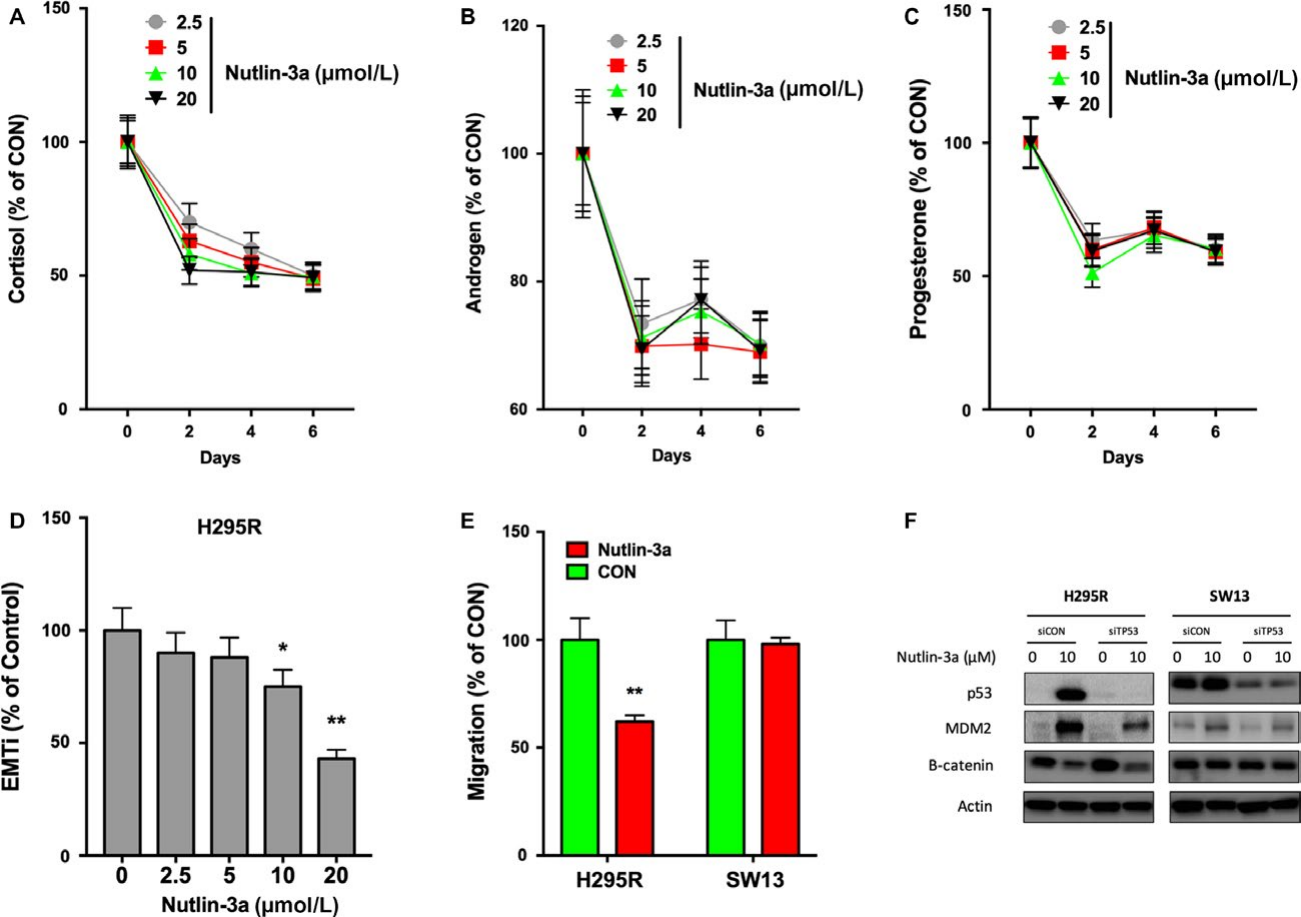
Nutlin‐3a inhibits hormone production and epithelial–mesenchymal transition (EMT) of ACC. Nutlin‐3a significantly inhibited production of (A) cortisol (B) androgen; and (C) progesterone in NCI‐H295R cells. Nutlin‐3a also significantly inhibited (D) EMT index and (E) cell migration of ACC. (F) Impact of TP53 status on Nutlin‐3a's effect on MDM2 and β‐catenin. (*P < 0.05, **P < 0.01).

High incidence of excessive hormone is a hallmark of ACC and is detrimental. By testing hormone levels in the media of H295R cells, we found that Nutlin‐3a induced a sharp decline in cortisol, androgen, and progesterone in ACC on day 2 of treatment (Fig. 4A–C). The hormone‐inhibitory effect was not dose‐dependent (Fig. 4A–C). EMT and migration were surrogates for metastatic potential. Using the EMTi, we observed significant inhibition of EMT by Nutlin‐3a at 10 *μ*mol/L or above (Fig. 4D). Nutlin‐3a also demonstrated selective inhibition of migration in H295R but not in SW13 cells (Fig. 4E). An exploratory mechanistic analysis was carried out and we found that Nutlin‐3a decreased β‐catenin level in H295R cells independent of TP53 status (Fig. 4F). Such effect was not present in SW13 cell even when *TP53* were silenced (Fig. 4F).

### Nutlin‐3a inhibits tumor growth in vivo

**Figure 5:**
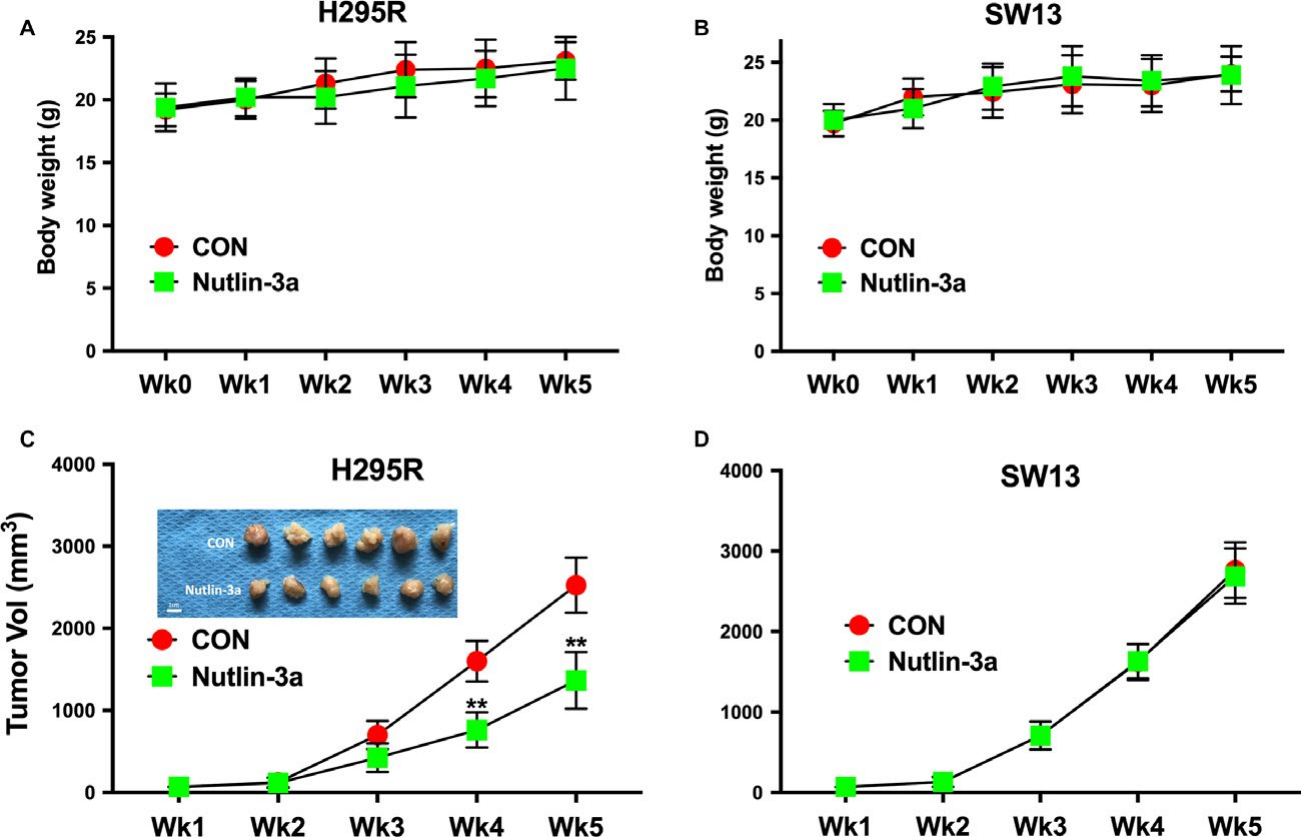
Nutlin‐3a selectively inhibited growth of ACC cell with *CTNNB1* mutation in vivo. Xenograft tumor model using H259R and SW13 cells showing tolerable toxicity in mice bearing (A) H295R and (B) SW13 cells; and selective inhibition in tumor growth by Nutlin‐3a in (C) H295R but not in (D) SW13 cells; Representative picture of tumor after 5 weeks of implantation. (**P < 0.01).

To investigate the effect of Nutlin‐3a on antitumor activities in vivo, we established xenograft tumor models with H295R and SW13 cells in nude mice. Toxicity profile demonstrated nonsignificant difference between mice treated with Nutlin‐3a or control (Fig. 5A and B). Assessment of tumor volume showed that the Nutlin‐3a‐treated groups had delayed tumor growth compared to the control group and the group treated with PBS control (Fig. 5C). Nutlin‐3a showed no inhibitory effect on SW13 cells (Fig. 5D). Together, we use in vivo and in vitro models to confirm that Nutlin‐3a selectively inhibits ACC cells with *CTNNB1* mutation.

## Discussion

Wnt/β‐catenin signaling plays a pivotal role in the formation of adrenal gland. Arising from a common adrenal–gonadal primordium, mesenchymal cells develop into adrenal precursor cells and bipotential gonadal precursor cells, which are two distinct population and develop into the adrenal cortex and the ovaries or testis, respectively 18. This effect is steroidogenic factor 1 (*SF1*)‐driven, and transcription of genes controlling adrenal–gonadal cell fate is regulated by Wnt/β‐catenin signaling cascades 19.

Likewise, alteration in Wnt/β‐catenin signaling plays a critical role in tumorigenesis of adrenal gland. *CTNNB1* mutation is the predominant alteration within Wnt/β‐catenin axis in ACC with consolidation from other genetic alterations in ZNRF3, APC, and MEN1. Most mutations in *CTNNB1* cluster on the N‐terminal segment of β‐catenin, also known as the β‐TrCP binding motif, including S45P, S45F, T41A, S45del, G34E, G34R, G34V, S45_P52del, A39Efs*3, and Y30* 9. Those activating mutations in this region render degradation and ubiquitinylation of β‐catenin inefficient, and accumulated β‐catenin can translocate into nucleus for continuous transcription of target genes.

In this study, we first explored the association between *CTNNB1* mutation and clinical presentations of ACC, namely hormone excess. We find that *CTNNB1* mutation is associated with excessive hormone but not with specific type of hormone. Specific mutations in adrenal gland, S33C, and G34R, S45P are reported to produce aldosterone in zona glomerulosa type of aldosterone‐producing adenoma 20. In our study, we find that two of the mutations are associated with a variety of hormone types in ACC patients in the TCGA cohort. Interestingly, S45P has previously been identified in adrenal tissue; the same residue is also mutated in H295R cells. The reason for different hormones produced with the same mutation type in adrenal tumors could root from tropism and cell context. *CTNNB1* mutation may need to occur in specific adrenocortical cell types for the full effects to be seen.

Although Wnt/β‐catenin signaling plays a broad and pivotal role in cancer development, Wnt‐targeting agent is currently lacking. Among different strategies to direct Wnt‐targeting anticancer therapy, exploring drugs previously approved for other diseases for a Wnt‐targeting activity is an actionable approach 21. In the current study, we identified Nutlin‐3a, a classic MDM2 inhibitor, with unexpected drug effect in *CTNNB1*‐mutated cancer cells. Intriguingly, theoretical drug resistance to *TP53* mutation is also found to be circumvented by concomitant *CTNNB1* mutation. We have also found that Nutlin‐3a inhibits several properties of H295R cells, including proliferation, EMT, hormone production, and tumorigenesis. The results in‐silico together with our validation in vitro and in vivo, render Nutlin‐3a an attractive pharmaceutical agent for *CTNNB1*‐mutant ACC. Indeed, we speculate that the selectivity for Nutlin‐3a in *CTNNB1*‐mutant cancer cells is cancer type‐specific. Although data reproduced from GDSC database show significant selectivity toward *CTNNB1*‐mutant cells, there are some outliers that exhibit insensitivity. Also, disparity between our findings on SW13 and H295R cells supports the notion, as those cells represent different types of adrenocortical cancer. Our findings also raise questions regarding crosstalk between canonical Wnt signaling and MDM2, which are previously suggested to be very loosely connected 22. Scholars suggest disruption of the RP‐MDM2‐p53 pathway accelerates APC loss‐induced colorectal tumorigenesis, yet how much APC loss can simulate *CTNNB1* mutation in the ACC context remains undetermined. We also investigated associations between genetic alterations of *CTNNB1*,* TP53*, and *MDM2* in TCGA cohort at expressional, mutational, and copy number levels yet no significant association is noted. In ACC, both *CTNNB1* and *TP53* mutations are considered truncal and irrelevant to whole genome doubling. Our findings may indicate that Wnt/β‐catenin effects downstream of TP53/MDM2. We suggest that mutated or functionally enhanced *CTNNB1* requires MDM2 to exert tumorigenic effect regardless of *TP53* status, proposing a novel nexus between Wnt/β‐catenin and p53 signaling. Previous reports suggest that Wnt/β‐catenin signaling regulates the proliferation and differentiation of mesenchymal progenitor cells through the p53 pathway 23. Also, p53 and microRNA‐34 are suggested to be suppressors of canonical Wnt/β‐catenin signaling 24. However, those reports provided indirect evidence that solely account in part for our observation and can hardly be extrapolated to explain the sensitivity of *CTNNB1* mutation to Nutlin‐3a. In vitro mechanistic studies are currently undergoing to further address the issue.

## Conclusion

Adrenocortical carcinoma (ACC) is a rare endocrine malignancy that is highly aggressive and lacks definitive treatment. We found that Nutlin‐3a selectively inhibited the growth of ACC cell with *CTNNB1* mutation in vitro and in vivo. Several new derivatives of Nutlin‐3a has now entered clinical trials, holding promise for targeted MDM2 inhibition in *CTNNB1*‐mutant ACC. However, how p53/MDM2 axis coordinate with Wnt/β‐catenin signaling in ACC warrants further study.

## Conflict of Interest

None declared.
